# Exosomes derived from human umbilical cord mesenchymal stem cells ameliorate IL-6-induced acute liver injury through miR-455-3p

**DOI:** 10.1186/s13287-020-1550-0

**Published:** 2020-01-23

**Authors:** Mingyang Shao, Qing Xu, Zhenru Wu, Yuwei Chen, Yuke Shu, Xiaoyue Cao, Menglin Chen, Bo Zhang, Yongjie Zhou, Rong Yao, Yujun Shi, Hong Bu

**Affiliations:** 1 Laboratory of Pathology, Key Laboratory of Transplant Engineering and Immunology, NHC, West China Hospital, Sichuan University, 37 Guoxue Road, Chengdu, 610041 China; 2 Sichuan Stem Cell Bank & Sichuan Neo-Life Stem Cell Biotech Inc., Chengdu, 610037 China; 3 The Emergency Department, West China Hospital, Sichuan University, Chengdu, 610041 China; 4 Department of Pathology, West China Hospital, Sichuan University, Chengdu, 610041 China

**Keywords:** Exosome, microRNA-455-3p, hUC-MSCs, Inflammation, Acute liver injury

## Abstract

**Background:**

Using a toxin-induced nonhuman primate model of acute liver failure (ALF), we previously reported that peripheral infusion of human umbilical cord mesenchymal stem cells (hUC-MSCs) strongly suppresses the activation of circulating monocytes and interleukin-6 (IL-6) production, thereby disrupting the development of a cytokine storm and improving the prognosis of monkeys. MSCs are considered to play a therapeutic role under different stresses by adaptively producing specific factors, prompting us to investigate the factors that hUC-MSCs produce in response to high serum levels of IL-6, which plays a critical role in initiating and accelerating ALF.

**Methods:**

We stimulated hUC-MSCs with IL-6, and the hUC-MSC-derived exosomes were deeply sequenced. The miRNAs in the exosomes that have potential to suppress IL-6-associated signaling pathway were screened, and the role of one of the most possible miRNAs was tested in the mouse model of inflammatory liver injury.

**Result:**

We determined that miR-455-3p, which is secreted through exosomes and potentially targets PI3K signaling, was highly produced by hUC-MSCs with IL-6 stimulation. The miR-455-3p-enriched exosomes could inhibit the activation and cytokine production of macrophages challenged with lipopolysaccharide (LPS) both in vivo and in vitro. In a chemical liver injury mouse model, enforced expression of miR-455-3p could attenuate macrophage infiltration and local liver damage and reduce the serum levels of inflammatory factors, thereby improving liver histology and systemic disorder.

**Conclusions:**

miR-455-3p-enriched exosomes derived from hUC-MSCs are a promising therapy for acute inflammatory liver injury.

## Background

Liver damage caused by a variety of etiologies can lead to acute liver injury and even acute liver failure (ALF). Patients often die of hepatic encephalopathy, coagulopathy, multiple organ failure (MOF), etc. [1]. Previously, we established an amanitin-induced acute liver failure model in monkeys, which comprehensively revealed the pathophysiological process of ALF [2]. After the induction of toxins, the initial injury of the liver is not serious, but a secondary uncontrolled systemic inflammatory response syndrome (SIRS) is the leading cause of multiple organ failure and death in animals [2]. Most strikingly, bone marrow-derived circulating monocytes are activated, characterized by overexpression of IL-6, before the occurrence of a cytokine storm and significant liver damage. Subsequently, IL-6 becomes an important initiator of an uncontrolled cytokine storm [3]. At the same time, many circulating monocytes migrate to the liver and differentiate into mature Kupffer cells that aggravate the local liver damage, and as a result, ALF occurs. In monkey and mouse toxic liver injury models, suppression of monocyte activation, especially inhibition of IL-6 secretion, can effectively control the occurrence of a cytokine storm, ameliorate liver damage, and improve animal survival.

Mesenchymal stem cells (MSCs) are tissue stem cells that are widely distributed in various tissues, such as the bone marrow, liver, fat, placenta, and umbilical cord. MSCs have multidirectional differentiation potential. They also secrete various growth factors and cytokines that promote cell regeneration, tissue repair, and immune regulation. MSCs have been used in mouse models for the treatment of a variety of diseases, including acute lung injury, myocardial infarction, diabetes, sepsis, liver dysfunction, and acute renal failure [4, 5]. Since MSCs have shown promising clinical application prospects in animal models of human diseases, MSC-based therapies have been carried out in multiple clinical trials for the treatment of various disorders [6–8]. As shown on www.ClinicalTrials.gov, dozens of MSC-based clinical therapies are underway for different types of liver injury, including autoimmune hepatitis, alcoholic liver cirrhosis, and liver fibrosis. However, MSC-based therapies have been strongly limited by their unclear mechanism of action [5, 9].

We previously established a toxin-induced nonhuman primate model of ALF [2]. In this model, we found that early peripheral infusion of human umbilical cord mesenchymal stem cells (hUC-MSCs) significantly rescued the monkeys from lethal ALF. Mechanistically, hUC-MSCs were not found to directly differentiate into hepatocytes, promote liver regeneration, or regulate T cells and B cells. However, early treatment of hUC-MSCs strongly inhibited the overproduction of IL-6 by reducing the activation of circulating monocytes, thus significantly inhibiting cytokine storms and improving liver damage and animal survival [10]. Furthermore, in vitro experiments revealed that coculture of hUC-MSCs inhibited LPS-induced macrophage activation and inflammatory factor secretion [10]. However, the mechanism by which hUC-MSCs inhibit macrophage activation remains unclear.

Exosomes are monolayer membrane vesicles actively secreted by cells into the extracellular space. They have a lipid bilayer membrane structure with a diameter of approximately 50–150 nm and contain diverse bioactive substances, such as transcription factors, oncogenes, miRNAs, lncRNAs, and mRNAs [11].. The exosome membrane protects these molecules from degradation before they reach the target cells (receptor cells) [12]. Exosomes can be engulfed by different cells, such as macrophages, endothelial cells, and tumor cells [13, 14]. Donor cell-derived exosomes directly activate cell surface receptors via proteins and biologically active lipid ligands and deliver their effectors to recipient cells. These foreign effectors also perform their own distinct functions in the recipient cells; for example, transcription factors can directly initiate gene transcription, mRNA can be translated into proteins, and miRNAs and lncRNAs can regulate gene transcription and translation. Therefore, exosomes are an important means of intercellular communication and play important roles in immune responses, immune regulation, inflammatory responses, and stem cell phenotypic transformation [15–18].

miRNAs are small noncoding RNAs that serve as regulators of mRNA expression and translation efficiency in most cell types. miRNAs contain a 6- to 8-nucleotide seed sequence corresponding to the complementary sequence in the 3′-UTR of the target mRNAs [19]. Binding of miRNA to mRNA results in the targeted recruitment of mRNA to the RNA-induced silencing complex (RISC), leading to translational arrest and mRNA degradation. Through these mechanisms, miRNAs reduce the protein expression of target mRNAs [19]. Interestingly, miRNAs are mainly delivered to the extracellular body in the form of exosomes, which are ubiquitous in the peripheral blood and cell culture medium. The exosome membrane effectively protects miRNAs from degradation by RNase [19, 20]. In turn, miRNAs are also one of the most important effectors of exosomes. After receiving miRNA-enriched exosomes, gene expression of the receptor cell has the potential to be regulated by the foreign miRNAs [20]. Therefore, miRNA-enriched exosomes play an important role in cell-to-cell communication [21, 22].

Recent studies suggest that the protective effects of MSCs on tissue repair and immune regulation may be mainly achieved by paracrine effects [23]. MSC-derived exosomes have shown therapeutic effects in models of myocardial ischemia, acute lung injury/ischemia, and skin trauma [24, 25]. In addition, cultures containing MSC-derived exosomes have therapeutic effects in a number of preclinical models [8, 26]. Similar to MSC-based therapy, MSC-derived exosomes are also beneficial for liver diseases such as liver fibrosis, drug-induced liver injury, and liver ischemia/reperfusion injury [27]. Due to their wide plasticity, MSCs play different roles in different pathophysiological environments, which is a prerequisite for the successful treatment of various diseases [28]. We speculate that in the early stage of toxic liver injury, the rapidly increased IL-6 is one of the first internal stresses that infused hUC-MSC encounter. Therefore, in this study, to mimic the situation in vivo, we stimulated hUC-MSCs with IL-6 in vitro and determined that miR-455-3p-enriched exosomes derived from hUC-MSCs have a promising therapeutic effect on acute liver injury by inhibiting the overactivation of monocytes and macrophages.

## Materials and methods

### Animals and treatment

All mouse experimental procedures were approved by the Animal Care and Use Committee of Sichuan University. Eight-week-old male mice (C57BL/6) were purchased from Chengdu Dossy Experimental Animals Co., Ltd. To establish an acute liver injury model or endotoxemia model, the mice were intraperitoneally injected with 10% carbon tetrachloride (CCl4) at 10 mL/kg or LPS at 3 mg/kg. Mice were sacrificed at the indicated time points for tissue and blood harvest.

### Cell lines and treatment

Human umbilical cords were donated by women who underwent cesarean sections. Informed consent was obtained from the subjects’ families. hUC-MSCs were collected at the Sichuan Stem Cell Bank, Chengdu, China, and cultured with serum-free medium (Gibco, USA). hUC-MSCs were identified by flow cytometry as described in previous studies [15]. The THP-1 cell line was obtained from Cellcook Biotech Guangzhou Ltd., and the cell line was authenticated by short tandem repeat (STR) profiling. The cells were identified as macrophages by immunohistochemical staining of CD68 (Abcam, USA) after induction with 100 μg/mL phorbol myristate acetate (PMA, Sigma-Aldrich, St. Louis, MO, USA) [20].

For the coculture system, hUC-MSCs (10^5^/well) were cultured in the upper chamber of a Transwell plate (0.4-μm polycarbonate filter, Corning), and macrophages were grown in the lower chamber and stimulated with LPS (100 ng/mL, Sigma-Aldrich). The medium and cells were collected after 48 h. To inhibit exosome secretion, hUC-MSCs were pretreated with 10 μM GW4869 (MCE, Monmouth Junction, NJ, USA) for 24 h.

### Exosome purification and characterization

hUC-MSCs were cultured with 1 ng/mL IL-6 (Sigma-Aldrich) in the medium for 48 h, and the supernatant was collected for exosome purification using an ExoQuick ULTRA EV isolation kit (SBI, Palo Alto, CA, USA). The characterization of exosomes was confirmed by electron microscopy and particle size by NanoSight analysis (Shanghai Oe Biotech Co., Ltd.) as described [19]. The expression of the exosome-specific marker TSG101 (SBI) and the EV-related protein markers CD63 and CD81 (SBI) were analyzed by western blotting. miRNAs were extracted from exosomes using TRIzol reagent (Invitrogen, USA) for qPCR analysis or sequencing at Shanghai Oe Biotech. To identify the transport of exosomes, exosomes were labeled with PKH26 fluorescent dye (Sigma-Aldrich) [19].

### Cytokine analysis

A MILLIPLEX MAP Human Cytokine/Chemokine kit (Millipore, Billerica, MA, USA; cat # HCYTOMAG-60 K) was used to quantify the levels of cytokines, chemokines, and growth factors in human cell supernatants on a Luminex 200 System (Millipore) according to the manufacturer’s instructions. A MILLIPLEX MAP Mouse Cytokine/Chemokine kit (Millipore; cat # MCYTMAG-70 K-PX32) was used to quantify the levels of serum cytokines, chemokines, and growth factors in mice.

### Flow cytometry analysis

Antibodies were purchased from BioLegend (San Diego, CA, USA). The proportion of monocytes/macrophages in peripheral blood was identified using fluorescently labeled antibodies. Monocytes/macrophages were detected by staining for CD45, CD14, CD16, or CCR2 [20] and analyzed using CytExpert software.

### Statistical analysis

GraphPad Prism 7.0 was used for statistical analyses. All data are represented as the mean ± SEM. The statistical significance of the differences between various treatments was measured by either the two-tailed Student’s *t* test or one-way ANOVA with Bonferroni post-test. Differences were considered statistically significant when *p* < 0.05.

## Results

### hUC-MSC-derived exosomes inhibit macrophage activation

**Fig. 1:**
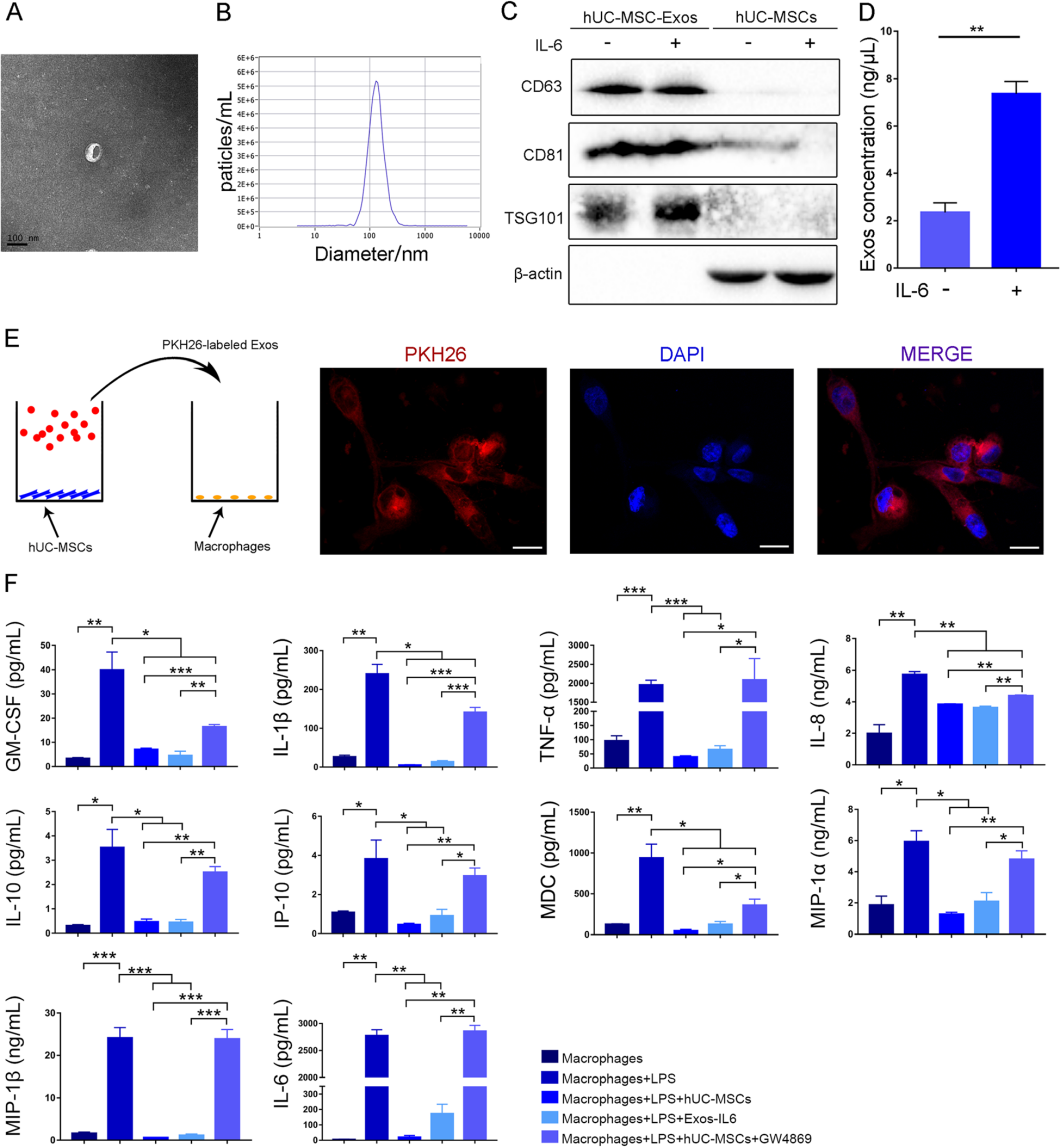
Exos-IL6 inhibit macrophage activation. **a** Electron microscopy analysis of vesicles secreted by hUC-MSCs (scale bar = 100 nm). **b** The particle size of the vesicles secreted by hUC-MSCs was measured by NanoSight analysis. **c** Exosome-specific markers TSG101 and CD63 and CD81 were measured by western blot analysis. **d** Exosome abundance in hUC-MSC medium with (Exos-IL6) or without IL-6 (Exos-NC). **e** PKH26-labeled exosomes were coincubated with macrophages and examined by confocal microscopy (scale bar = 20 μm). **f** Exos-IL6 were added to macrophages to detect inflammatory cytokines, chemokines, and growth factors in cell culture media. GM-CSF, granulocyte-macrophage colony-stimulating factor; IL, interleukin; MIP-1, macrophage inflammatory protein-1; IP-10, interferon-inducible protein-10; MDC, macrophage-derived chemokine; MCP-1, monocyte chemoattractant protein-1; TNF-ɑ, tumor necrosis factor. Data are presented as the mean ± SEM (error bar) of at least three independent experiments. *p < 0.05, **p < 0.01, and ***p < 0.001

We first tested whether hUC-MSCs can secrete exosomes under IL-6 stimulation. hUC-MSCs at passage 3 were cultured with 1 ng/mL IL-6 for 48 h. After removing the dead cells and debris, an ExoQuick ULTRA EV isolation kit was used to purify exosomes from the serum-free medium. Electron microscopy and NanoSight analysis showed that the particles contained a large number of hUC-MSC-derived vesicles with a diameter of 50–150 nm (Fig. 1a, b). Western blot analysis confirmed that the vesicles did not express β-actin but expressed the exosome-specific protein markers CD63 (a four-transmembrane protein accumulated in the multivesicular body), CD81, and TSG101 (mainly present in the cytoplasm) (Fig. 1c). These observations indicated that the vesicles were MSC-derived exosomes (MSC-Exos). Notably, after IL-6 stimulation, hUC-MSCs could secrete approximately three times more exosomes (Exos-IL6) than hUC-MSCs without IL-6 stimulation (Exos-NC, negative control) (Fig. 1d).

To confirm that MSC-Exos could be taken up by macrophages (Additional file 1: Figure S1), we labeled MSC-Exos with PKH26, which is a fluorescent dye that binds to the phospholipid bilayer membrane [19]. After a 48-h incubation, the PKH26-labeled MSC-Exos were taken up by macrophages, and the cells displayed apparent red fluorescence under a fluorescence microscope (Fig. 1e).

Our previous in vitro study demonstrated that when cocultured with hUC-MSCs, LPS-stimulated macrophages tremendously decreased the production of inflammatory factors, especially IL-6 [2]. To validate that hUC-MSCs regulate macrophage activation by exosomes, Exos-IL6 were added into the culture medium. Similar to the results of coculture with hUC-MSCs (Additional file 1: Figure S2), Exos-IL6 also significantly inhibited the secretion of various inflammatory factors in LPS-stimulated macrophages (Fig. 1f). Interestingly, when we blocked the production of MSC-Exos with GW4869, the coculture of hUC-MSCs failed to significantly decrease the release of inflammatory factors by macrophages exposed to LPS (Fig. 1f). It seems that hUC-MSCs inhibit macrophage activation mainly in an exosome-dependent manner.

### IL-6 induces changes in miRNAs in MSCs-Exos

**Fig. 2:**
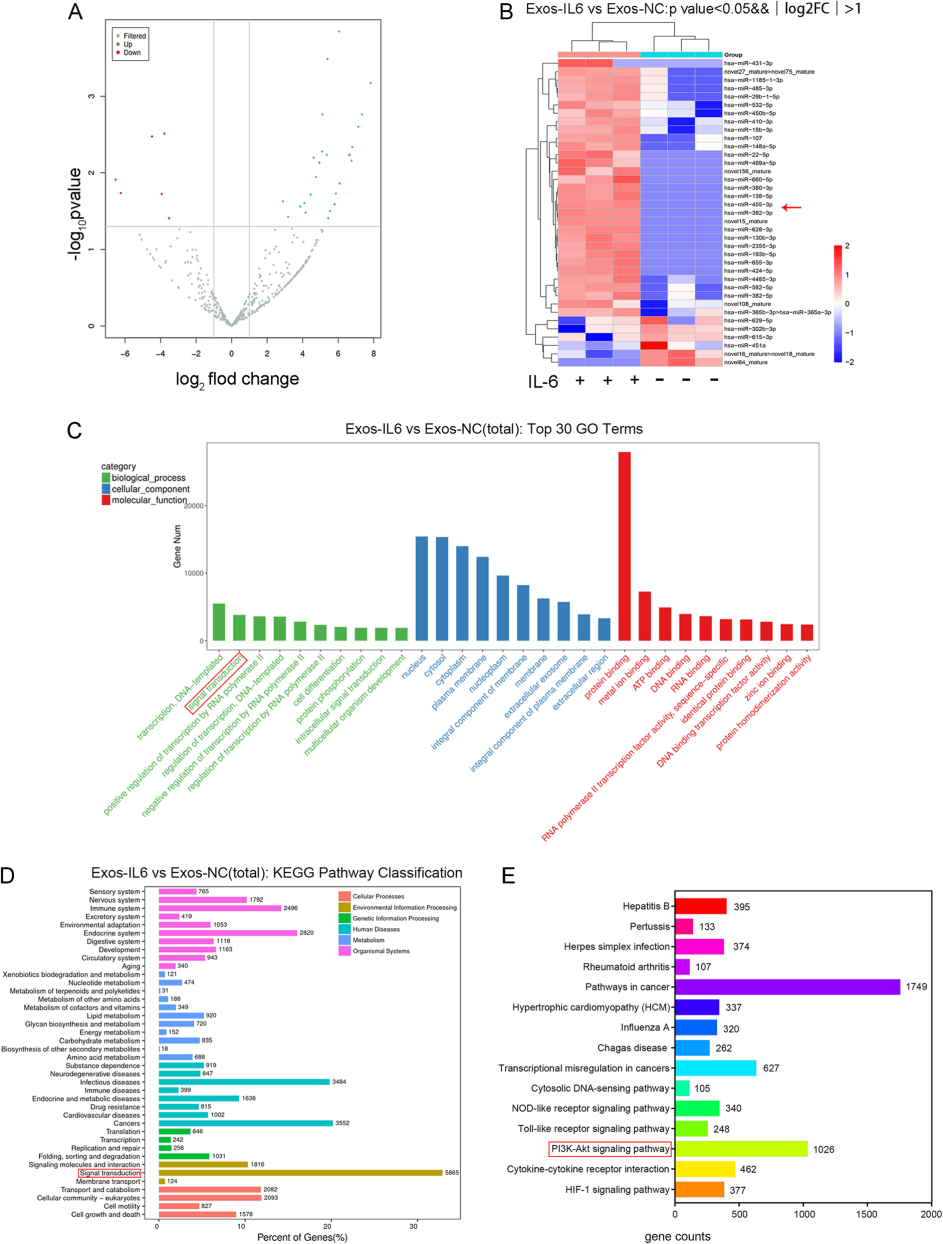
Analysis of differentially expressed miRNA between Exos-IL6 and Exos-NC. **a**Thirty-seven significantly differentially expressed miRNA volcanoes in Exos-IL6 (fold change > 2-fold; *p* < 0.05). **b** Heat map of differentially expressed miRNAs between Exos-IL6 and Exos-NC. **c** TOP 30 enriched GO terms of miRNA target genes. **d** The target genes of differentially expressed miRNAs were analyzed for KEGG pathway enrichment. **e** The number of target genes of differentially expressed miRNA target genes annotated under the IL-6-related signaling pathway

To assess IL-6-induced miRNA changes in MSC-Exos, we conducted deep sequencing of small RNAs extracted from Exos-IL6 and Exos-NC. We identified a set of miRNAs that were significantly differentially abundant between the two groups. In comparison with those in Exos-NC, 31 miRNAs were upregulated and 6 miRNAs were downregulated in Exos-IL6 (fold change > 2, *p* < 0.05) (Fig. 2a, b and Additional file 1: Table S1). The miRanda algorithm was used to match miRNA and mRNA sequences to predict the target relationship between miRNAs and mRNAs. We then conducted Gene Ontology (GO) analysis and Kyoto Encyclopedia of Genes and Genomes (KEGG) pathway analysis on the 17,589 predicted target mRNAs of the differentially expressed miRNAs. These target genes were found to be enriched in multiple signaling pathways (Fig. 2c, d). Among these signaling pathways, we focused on the signaling pathways involved in IL-6 and found that these miRNAs were widely involved in the regulation of IL-6-related signaling pathways, including pathways in cancers, the PI3K-Akt signaling pathway, cytokine-cytokine receptor interaction, and the Toll-like receptor signaling pathway (Fig. 2e). Among these upregulated miRNAs, five miRNAs, miR-455-3p, miR-424-5p, miR-485-3p, miR-431-3p, and miR-134-5p, were predicted to regulate the identified pathways.

**Fig. 3:**
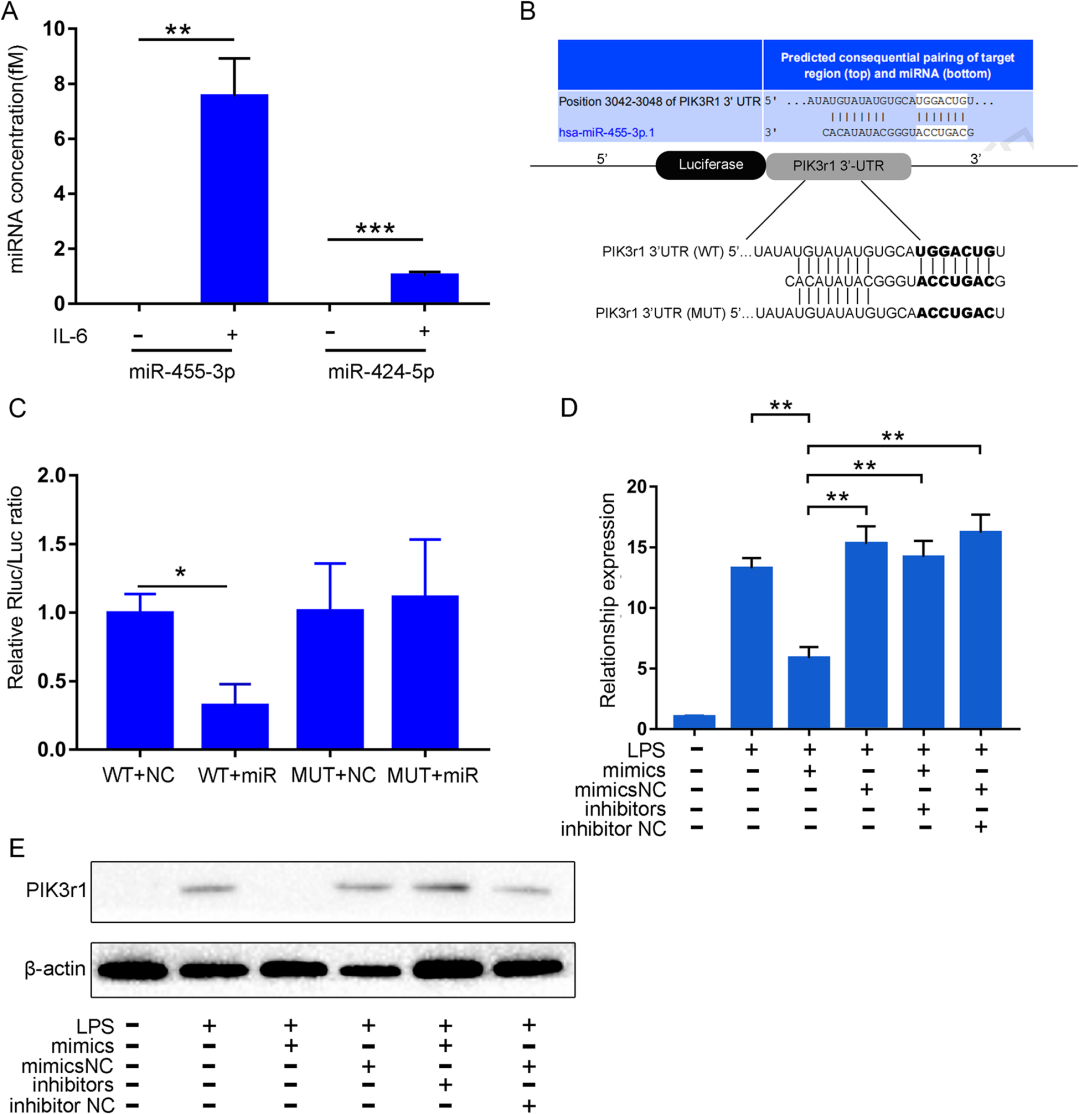
miR-455-3p in Exos-IL6 inhibits the expression of PIK3r1 in macrophages. **a** qPCR analysis of miR-455-3p and miR-424-5p expression levels in Exos-IL6 and Exos-NC. **b**, **c** Schematic diagram showing the putative miR-455-3p binding sites in PIK3r1. The sequences of wild-type PIK3r1 and mutant PIK3r1 are listed as well. Luciferase reporter gene assays were performed to measure the luciferase activity in macrophages. **d**, **e** Macrophages were transfected with miR-455-3p mimics, inhibitors, and their corresponding controls (mimics NC, inhibitors NC). qPCR assay and western blot were used to analyze the mRNA and protein levels of PIK3r1, respectively. Data are presented as the mean ± SEM (error bar) of at least three independent experiments. *p < 0.05, **p < 0.01, and ***p < 0.001

According to a fold change > 50, log_2_FoldChange > 5.5 and *p* < 0.01, we selected the upregulated miRNAs miR-455-3p and miR-424-5p to further validate the RNA sequencing results. Furthermore, absolute quantitative qPCR showed that the expression of miR-455-3p was more abundant (7.5 fM) than that of miR-424-5p (1 fM) (Fig. 3a). Therefore, we focused only on miR-455-3p in further studies.

### miR-455-3p inhibits macrophage activation by regulating PIK3r1

According to the results of TargetScan and miRanda analyses, we found that miR-455-3p may have a binding site in the 3′-UTR of the PIK3r1 gene (phosphoinositide-3-kinase regulatory subunit 1), which encodes p85α, a subunit of PI3K. PI3K is a well-known key factor in the activation of the IL-6-related signaling pathway [29, 30], and we speculated that miR-455-3p might negatively regulate IL-6 signaling by suppressing PI3K expression. To further demonstrate this, we performed a luciferase reporter assay. PIK3r1 cDNA was cloned into the Renilla luciferase gene (hRluc, pmiR-RB-REPORT h-PIK3R1-WT) and cotransfected with miR-455-3p or negative control miRNA (miR-NC) into macrophages (Fig. 3b). The results showed that the luciferase activity in the miR-455-3p-transfected macrophages was significantly reduced compared to the miR-NC group. The luciferase activity of the PIK3r1 cDNA-mutant vector was not significantly affected by miR-455-3p (Fig. 3c). In addition, qPCR and western blot analysis showed that miR-455-3p significantly decreased PIK3r1 expression both at the mRNA and protein levels in macrophages (Fig. 3d, e). These results indicated that miR-455-3p could inhibit macrophage activation by downregulating the target gene PIK3r1.

**Fig. 4:**
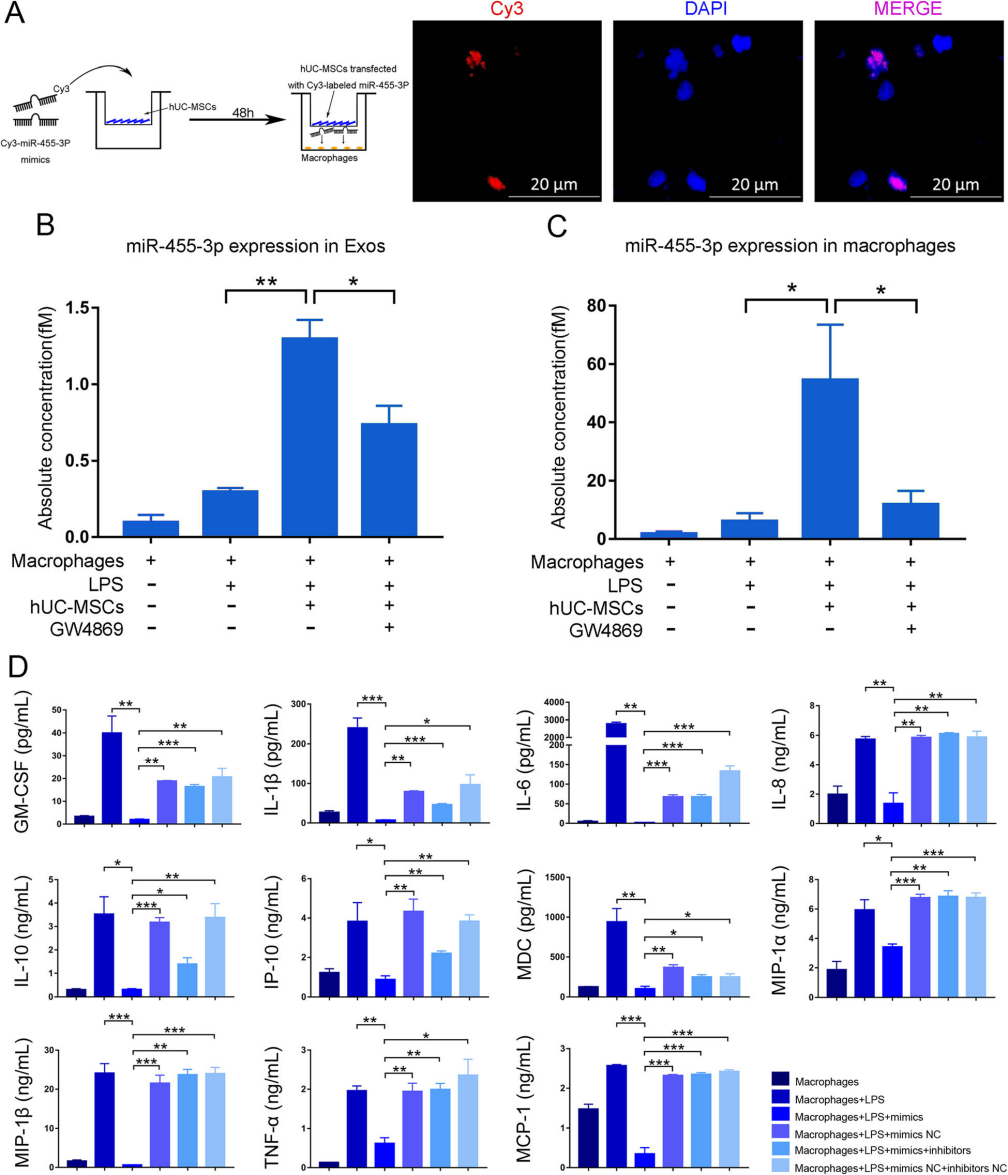
hUC-MSCs secrete miR-455-3p and transport it to macrophages to inhibit macrophage secretion of inflammatory factors. **a** hUC-MSCs transfected with Cy3-labeled miR-455-3p mimics were cocultured with macrophages in Transwell plates (membrane well = 0.4 μm). **b**, **c** Abundance of miR-455-3p in exosomes from cell culture medium and macrophages after coculture with hUC-MSCs. **d** After overexpression of miR-455-3p, the macrophage inflammatory factors were tested. Data are presented as the mean ± SEM (error bar) of at least three independent experiments. *p < 0.05, **p < 0.01, and ***p < 0.001

To determine whether hUC-MSCs transfer miR-455-3p to macrophages via exosomes, we performed miRNA tracing and coculture experiments. Cy3-labeled miR-455-3p mimics (purchased from Guangzhou RiboBio Co., Ltd., China) were transfected into hUC-MSCs. After removing the mimics that had not been transfected into cells, hUC-MSCs were cocultured with macrophages in a Transwell plate. The appearance of red fluorescent Cy3 dye in macrophages suggested that the Cy3-miR-455-3p mimics were delivered from the hUC-MSCs in the upper well to the macrophages in the lower well (Fig. 4a). Moreover, prior treatment with GW4869 blocked MSC-Exos production and delivery of miR-455-3p, and as a result, red fluorescence in the macrophages was significantly reduced (Additional file 1: Figure S3A). These results again indicated that miRNAs secreted by hUC-MSCs were taken up by macrophages (Figs. 1e and 4a).

We treated macrophages with LPS and examined the expression of miR-455-3p in the exosomes in the medium. In the presence of hUC-MSCs, miR-455-3p in the extracted exosomes was fourfold higher compared with that in the absence of hUC-MSCs (Fig. 4b). Moreover, miR-455-3p was significantly increased in the macrophages after coculture (Fig. 4c), suggesting the uptake of MSC-Exos by macrophages. Importantly, when hUC-MSCs were pretreated with GW4869 for 24 h, miR-455-3p was significantly decreased both in the medium and macrophages (Fig. 4b, c).

We next tested whether miR-455-3p alone had an inhibitory effect on macrophages. We transfected miR-455-3p mimics, inhibitors, and related controls into macrophages and cultured them for 48 h. As shown in Fig. 4d, miR-455-3p mimic transfection decreased all of the tested inflammatory factors produced by macrophages in response to LPS stimulation. When the miR-455-3p inhibitor was simultaneously added to the medium, the inhibitory effect of miR-455-3p was partially counteracted. Collectively, our data demonstrated that hUC-MSCs inhibited the activation of macrophages by secreting miR-455-3p-enriched exosomes.

### miR-455-3p inhibits macrophage activation in LPS-induced endotoxemia mice

We next investigated whether miR-455-3p inhibits macrophage activation in vivo. Before the injection of human cell-derived miR-455-3p in mice, we used TargetScan software to confirm that miR-455-3p had binding sites and an identical seed sequence in both the human and mouse PIK3r1 genes (Additional file 1: Figure S3B). These results indicated that miR-455-3p is highly conserved in humans and mice and might have similar functions in mice.

**Fig. 5:**
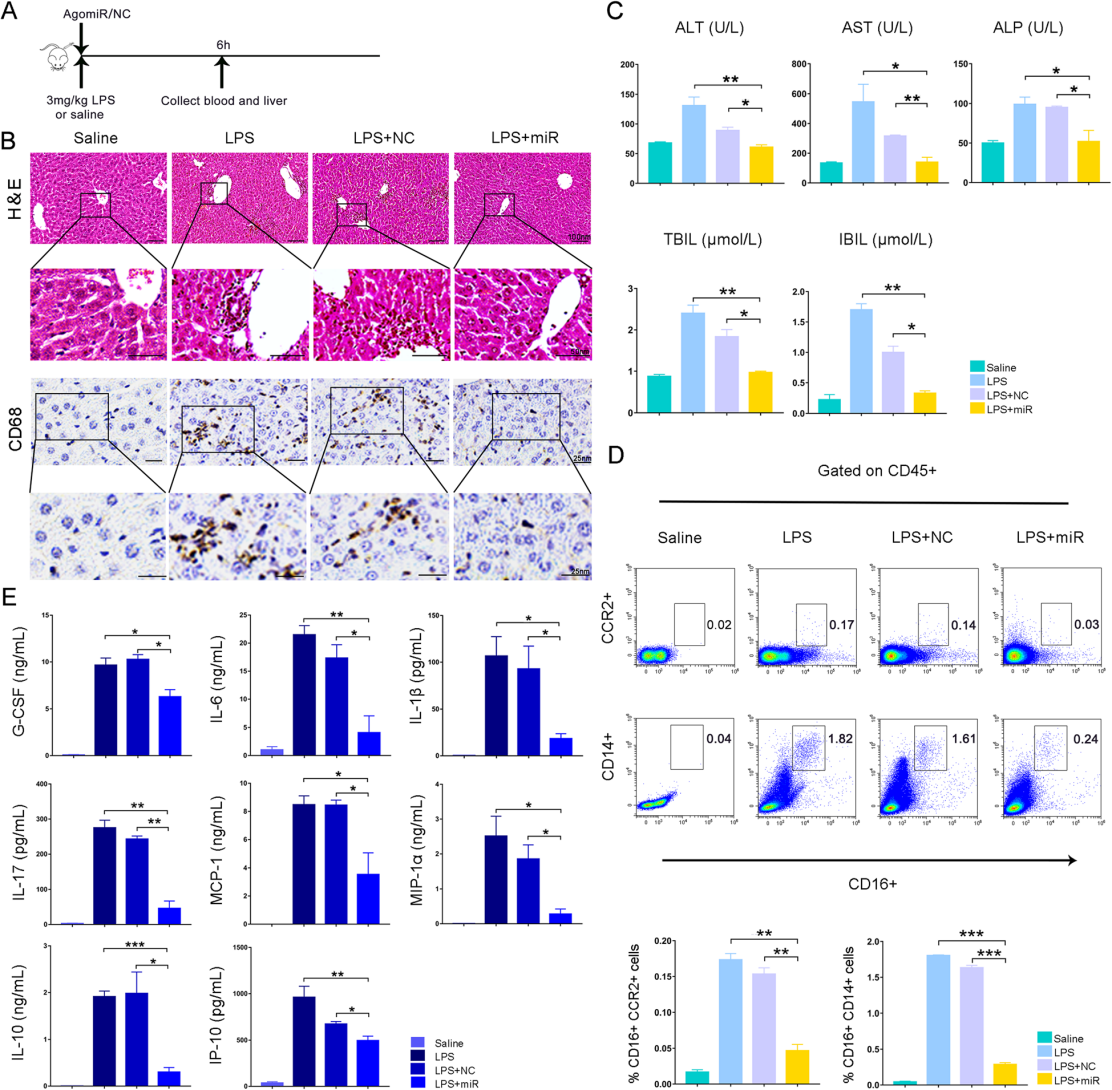
miR-455-3p treatment of LPS-induced endotoxemia mice. **a** Experimental procedure in mice. **b** The inflammatory infiltration in mouse tissues was visualized by H&E staining and CD68 immunohistochemistry. **c** Biochemical assays of hepatic indexes: alanine aminotransferase (ALT), glutamine-oxaloacetate transaminase (AST), indirect bilirubin (IBIL), alkaline phosphatase (ALP), and total bilirubin (TBIL). **d** Flow cytometry was used to analyze the activation rate of CD14 + CD16+ or CCR2 + CD16+ monocytes in CD45+ T cells. **e** The levels of inflammatory mediators in the blood were measured by Luminex. G-CSF, granulocyte colony-stimulating factor; IL, interleukin; MCP-1, monocyte chemoattractant protein-1; MIP-1ɑ, macrophage inflammatory protein-1ɑ; IP-10, interferon induction protein-10. Data are presented as the mean ± SEM (error bars). *N* = 5 mice per group; *p < 0.05, **p < 0.01, and ***p < 0.001. NC, miR-455-3p agomir negative control; miR, miR-455-3p agomir

By selectively activating monocytes/macrophages, particularly resident Kupffer cells in the liver, LPS has been widely used to generate an endotoxemia mouse model [19]. Mice were intraperitoneally injected with LPS (3 mg/kg). Then, miR-455-3p agomir or agomir negative control (agomir NC) was immediately injected via the tail vein. The mice were sacrificed for tissue and blood harvest 6 h later (Fig. 5a). After LPS injection, a large number of CD68-positive cells were observed in the liver, which indicated the activation of Kupffer cells (Fig. 5b). After treatment with miR-455-3p agomir, the number of CD68+ cells was significantly reduced (Fig. 5b). Biochemical assays of hepatic indexes showed a significant improvement in liver damage after miR-455-3p treatment (Fig. 5c). Flow cytometry assays demonstrated that after miR-455-3p treatment, the ratio of activated monocytes marked by CD14+CD16+ or CCR2+CD16+ was significantly lower than that in the agomir NC group (Fig. 5d). After LPS stimulation, proinflammatory macrophages can upregulate the levels of various inflammatory factors. We assessed the serum levels of inflammatory factors, and the results showed that most inflammatory factors were significantly increased after LPS stimulation, including G-CSF, IL-1β, IL-10, IL-17, IL-6, MCP-1, MIP-1α, and IP-10. miR-455-3p reduced the levels of inflammatory factors to varying degrees, especially IL-6 (Fig. 5e). Thus, in LPS-treated mice, miR-455-3p inhibited the activation of liver Kupffer cells and the secretion of inflammatory cytokines, leading to improved liver and systemic inflammatory responses.

### miR-455-3p improves CCl4-induced acute liver injury in mice

**Fig. 6:**
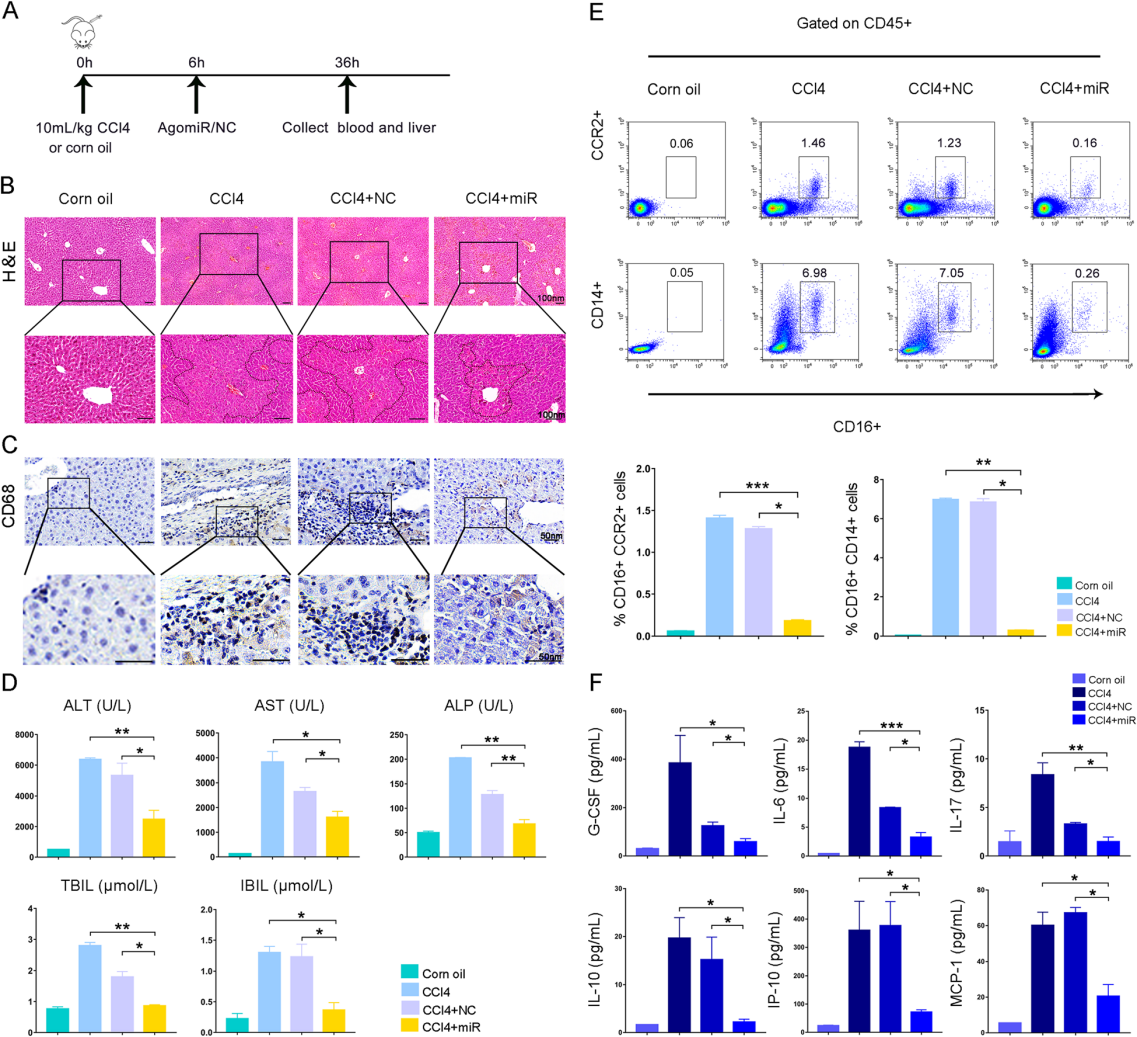
miR-455-3p is used to treat CCl4-induced acute liver injury in mice. **a** Experimental procedure in mice. **b** H&E staining of liver specimens. **c** CD68 immunohistochemistry. **d** Biochemical assays of hepatic indexes. **e** Flow cytometry analysis of the ratio of CD14 + CD16+ or CCR2 + CD16+ monocytes in peripheral blood CD45+ T cells. **f** Levels of inflammatory cytokines, chemokines, and growth factors in serum. Data are presented as the mean ± SEM (error bars). *N* = 5 mice per group; *p < 0.05, **p < 0.01, and ***p < 0.001. NC, miR-455-3p agomir negative control; miR, miR-455-3p agomir

We next established an acute chemical liver injury mouse model and investigated whether miR-455-3p improved liver damage by inhibiting monocytes/macrophages activation. Six hours after intraperitoneal injection of CCl4, miR-455-3p agomir or agomir NC was injected via the tail vein. The mice were sacrificed 36 h later (Fig. 6a). Histological examination showed that after CCl4 treatment, the hepatocytes exhibited extensive swollen and eosinophilic changes, and patchy necrosis of hepatocytes was prominent in the portal area (Fig. 6b). Immunohistochemistry staining showed marked infiltration of CD68+ Kupffer cells in the liver, indicating the activation of liver macrophages (Fig. 6c). Treatment with miR-455-3p apparently reduced the edema and necrosis of hepatocytes and the infiltration of CD68+ cells (Fig. 6b, c). Biochemical assays showed that miR-455-3p agomir treatment significantly decreased the serum levels of hepatic indexes, including ALT, AST, and bilirubin (Fig. 6d). Flow cytometry analysis also showed that miR-455-3p agomir treatment decreased the proportion of CD14 + CD16+ or CCR2 + CD16+ monocytes in the peripheral blood (Fig. 6e). As expected, the serum levels of inflammatory factors, such as IL-6, G-CSF, IL-17, IL-10, IP-10, and MCP-1, were significantly decreased in response to miR-455-3p agomir infusion (Fig. 6f).

## Discussion

Our previous studies showed that hUC-MSC therapy significantly improved ALF progression and prognosis in monkeys by suppressing circulating monocyte activation and IL-6 secretion; however, the crosstalk between hUC-MSCs and monocytes was still unclear. In this study, we revealed that hUC-MSCs produced a large amount of miR-455-3p-enriched exosomes in response to IL-6 stimulation. These exosomes inhibited the release of IL-6 as well as dozens of other inflammatory factors by LPS-challenged macrophages by targeting PIK3r1. In mouse models of endotoxemia and chemical liver injury, overexpression of miR-455-3p attenuated liver damage, macrophage infiltration, and serum levels of inflammatory factors. Therefore, hUC-MSCs might suppress the overactivation of monocytes/macrophages and improve liver damage and systemic homeostasis by secreting miR-455-3p-enriched exosomes.

Although MSC-based therapy has been widely regarded as a promising solution for various human diseases, it has been largely hindered by the unclear therapeutic mechanism, partly due to the lack of a strategy to trace the infused cells in vivo. One of the prominent features of MSCs is their strong plasticity to the varieties and quantities of local microenvironmental stimulatory factors, which helps MSCs play diverse roles in different circumstances [28]. In monkeys treated with amatoxin, exogenous hUC-MSCs encounter sharply increased IL-6 in the serum, which is one of the major changes in the internal environment. In this study, we initially simulated such stress environment with high IL-6 in vitro and found that the number of exosomes secreted by hUC-MSCs increased. Similar to hUC-MSCs, these exosomes inhibited LPS-challenged macrophages from secreting inflammatory factors, which, together with the finding that blocking exosome secretion failed to inhibit macrophage activation, suggested that hUC-MSCs suppress macrophages in an exosome-dependent manner.

Exosomes are important mediators of intercellular communication via their enclosed bioactive substances. After deep sequencing, we determined that miR-455-3p from hUC-MSCs might be one of the critical miRNAs that inhibits the activation of macrophages. Although NCBI and other publicly available databases indicate that miR-455-3p can be used as a marker for certain diseases, such as hepatocellular carcinoma, anaplastic large cell lymphoma, Alzheimer’s disease, and cerebrospinal fluid [29–32], there are no related reports showing that miR-455-3p is an anti-inflammatory factor. We found that miR-455-3p was able to suppress monocyte/macrophage activation, reduce the secretion of inflammatory factors, and significantly improve chemical liver injury. Bioinformatics analysis and luciferase reporter assay revealed that miR-455-3p directly targets the 3′-UTR of PIK3r1, which was further confirmed by the finding that overexpression of miR-455-3p significantly downregulated PIK3r1 expression at both the mRNA and protein levels. PIK3r1 is the regulatory subunit of the well-known factor phosphoinositide 3 kinase (PI3K) [33]. PI3K, as a positive regulator of IL-6, plays a key role in LPS-induced cytokine production [34, 35]. Therefore, miR-455-3p is likely to block the activation of the IL-6 signaling pathway by targeting the PIK3r1 gene.

Other reasons that limit the clinical application of stem cells include allogeneic rejection, tumorigenicity, and the tedious process of preparation and quality control. In contrast, MSC-derived exosomes can overcome the key limitations of treatment with cells. Compared with cells, exosomes have a tough lipid bilayer that enables them to be more stable [36]. Exosomes have the ability to penetrate deep tissues and avoid immune attack, which facilitates their delivery of therapeutic substances directly into target cells [37]. In this experiment, MSC-Exos could be absorbed by macrophages in vitro and act in a similar therapeutic effect as hUC-MSCs. Moreover, many in vivo studies have shown that MSC-derived exosomes can enter the liver [38], which indicates that MSC-Exos-based treatment is a promising alternative to MSC treatment, particularly in liver diseases.

There are still some limitations in the treatment with MSC-derived exosomes, including batch differences, purity, drug delivery, and off-target effects [39]. Therefore, it is particularly important to elucidate the effective components in exosomes. miRNAs are one of the most abundant components and the major bioactive substance in the exosome. Compared with exosomes that contain a great number of bioactive substances and have many unclear functions, miRNAs are more specific in function and easier to handle, making them more likely to be developed as industrialized pharmaceuticals. We demonstrated that miR-455-3p, which was markedly increased in exosomes derived from IL-6-challenged hUC-MSCs, was able to inhibit monocyte/macrophage activation both in vitro and in vivo; therefore, miR-455-3p is a promising candidate for the treatment of acute liver injury as well as other disorders characterized by the overactivation of macrophages.

There are several limitations in this study. First, IL-6 was used to simulate the inflammatory environment, and hUC-MSCs were expected to make adaptive changes and produce specific miRNA-enriched exosomes in this microenvironment. Although IL-6 is a critical factor that triggers and accelerates the inflammatory cascade, IL-6 is not the only factor evolved in this process. Stimulating hUC-MSCs with inflammatory serum might more realistically simulate the microenvironment. However, there are numerous exosomes in the serum, which hinders the identification of the hUC-MSC-derived exosomes. Second, miR-455-3p is just one of the significantly increased miRNAs in exosomes. Although miR-455-3p strongly inhibits monocyte/macrophage activation and improves acute liver injury, the roles of other miRNAs deserve further investigation. Studies have reported that one miRNA can target several genes and that one gene can be regulated by different miRNAs. The side effects of miR-455-3p are still unclear. Whether there are more effective miRNAs that inhibit IL-6 signaling still requires further investigation.

## Conclusions

hUC-MSC-derived miR-455-3p-enriched exosomes are able to suppress monocyte/macrophage activation and alleviate acute liver injury by inhibiting IL-6-related signaling pathways. Our study provides a basis for miR-455-3p as a treatment for acute inflammatory liver injury.

## Supplementary information

**Additional file 1: Figure S1.** Identification of the cell morphology of hUC-MSCs cells and macrophages. **Figure S2.** hUC-MSCs inhibit macrophage secretion of inflammatory factors. **Figure S3.** (A) The fluorescence results of hUC-MSCs pretreated with GW4869 and cocultured with macrophages. (B) The key sequences of human and murine miR-455-3p were basically identical. **Table S1.** After deep sequencing of Exos-IL6 and Exos-NC, 31 miRNAs were found to be upregulated and 6 miRNAs were downregulated in the Exos-IL6 group (fold change > 2, *p* < 0.05).

## Data Availability

All data generated or analyzed during this study are included in this published article.

## Abbreviations

ALF: Acute liver failure
ALP: Alkaline phosphatase
ALT: Alanine aminotransferase
AST: Glutamine-oxaloacetate transaminase
CCl4: Carbon tetrachloride
Exos-IL6: hUC-MSCs with IL-6 stimulation
Exos-NC: hUC-MSCs without IL-6 stimulation
GM-CSF: Granulocyte-macrophage colony-stimulating factor
GO: Gene Ontology
hUC-MSCs: Human umbilical cord mesenchymal stem cells
IBIL: Indirect bilirubin
IL: Interleukin
IL-6: Interleukin-6
IP-10: Interferon-inducible protein-10
KEGG: Kyoto Encyclopedia of Genes and Genomes
LPS: Lipopolysaccharide
MCP-1: Monocyte chemoattractant protein-1
MDC: Macrophage-derived chemokine
MIP-1: Macrophage inflammatory protein-1
MOF: Multiple organ failure
MSC-Exos: MSC-derived exosomes
MSCs: Mesenchymal stem cells
PI3K: Phosphoinositide 3-kinase
RISC: RNA-induced silencing complex
SIRS: Systemic inflammatory response syndrome
STR: Short tandem repeat
TBIL: Total bilirubin
TNF-ɑ: Tumor necrosis factor
