# Supplementary material for: Exosomes derived from human umbilical cord mesenchymal stem cells ameliorate IL-6-induced acute liver injury through miR-455-3p
Source: Stem Cell Res Ther. 2020 Jan 23;11:37. doi: 10.1186/s13287-020-1550-0 (PMC6979401; doi:10.1186/s13287-020-1550-0)
Supplement: Supplementary file 1 — Additional file 1: Figure S1. Identification of the cell morphology of hUC-MSCs cells and macrophages. Figure S2. hUC-MSCs inhibit macrophage secretion of inflammatory factors. Figure S3. (A) The fluorescence results of hUC-MSCs pretreated with GW4869 and cocultured with macrophages. (B) The key sequences of human and murine miR-455-3p were basically identical. Table S1. After deep sequencing of Exos-IL6 and Exos-NC, 31 miRNAs were found to be upregulated and 6 miRNAs were downregulated in the Exos-IL6 group (fold change > 2, p < 0.05). [file 13287_2020_1550_MOESM1_ESM.docx]

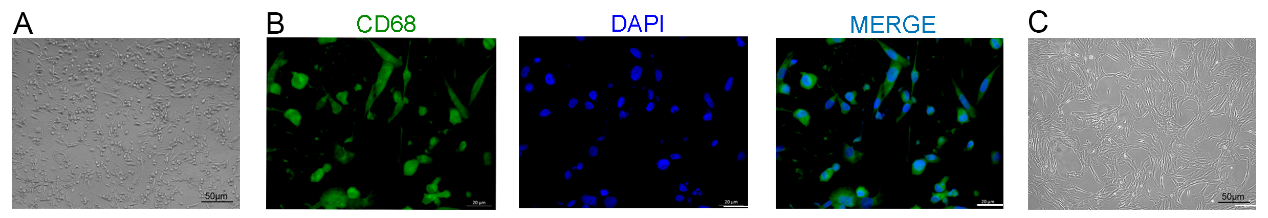


**Figure S1 Identification of the cell morphology of hUC-MSCs cells and macrophages.** (A) hUC-MSC morphology. (B) PMA-induced THP-1 cells act as macrophages. (C) CD68 staining confirmed that the induced THP-1 cells were macrophages.


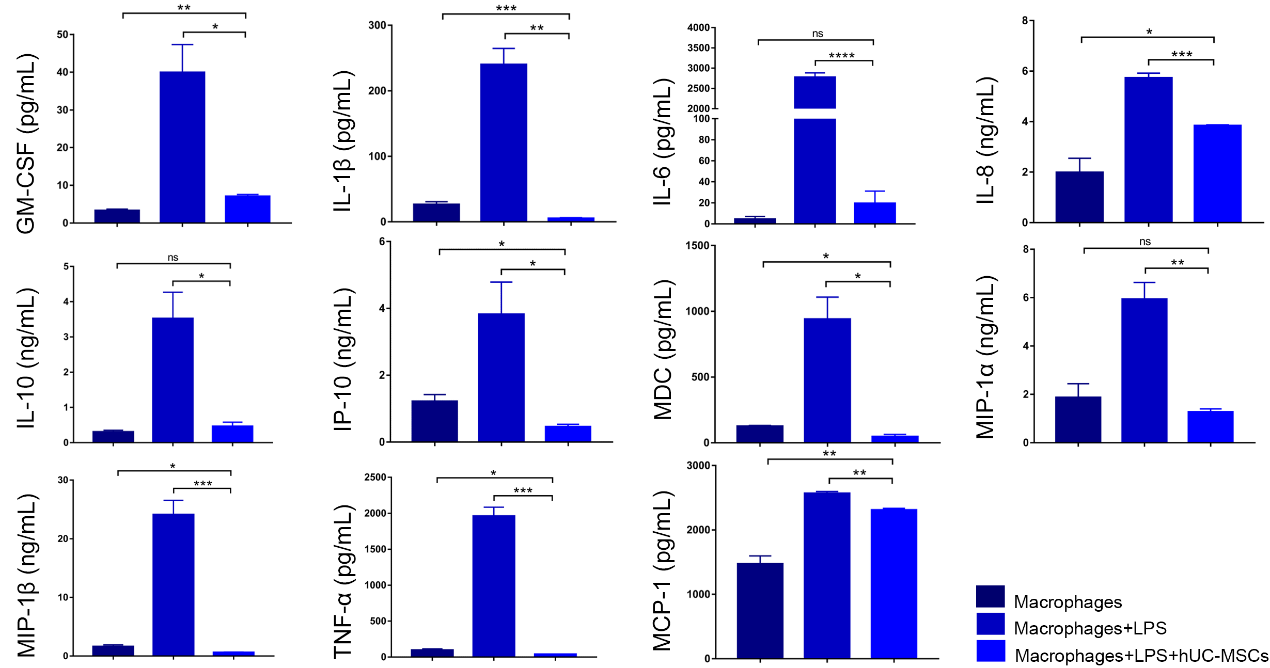


**Figure S2 hUC-MSCs inhibit macrophage secretion of inflammatory factors.** After coculture of hUC-MSCs with macrophages, we examined the levels of inflammatory factors in the cell culture medium. Data are presented as the mean ± SEM (error bars) of at least three independent experiments. *p < 0.05, **p < 0.01, and *** p <0.001.


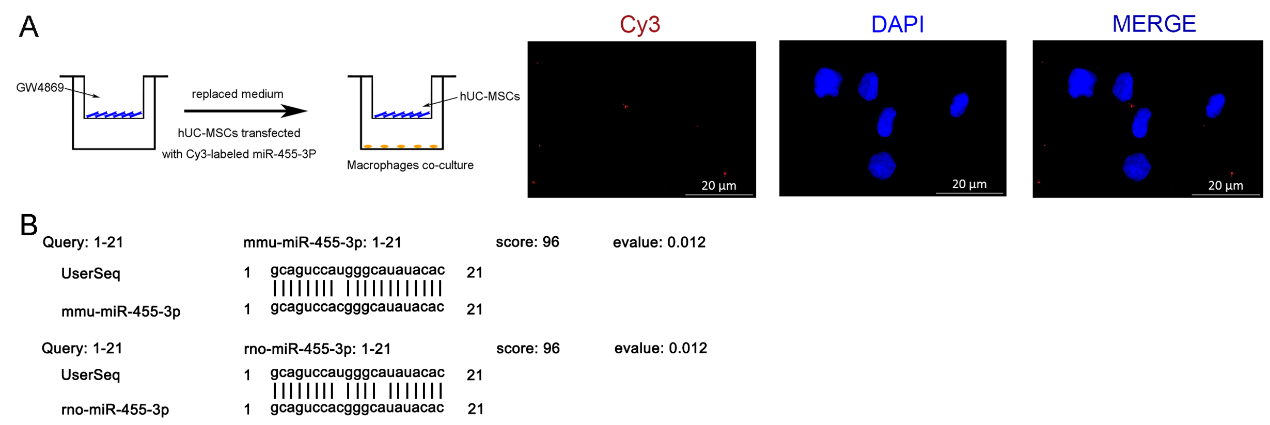


**Figure S3** (A) The fluorescence results of hUC-MSCs pretreated with GW4869 and cocultured with macrophages. (B) The key sequences of human and murine miR-455-3p were basically identical.

| Up-regulated miRNA_id | Sequence | Length |
| --- | --- | --- |
| hsa-miR-455-3p | GCAGTCCATGGGCATATACAC | 21 |
| hsa-miR-380-3p | TATGTAATATGGTCCACATCTT | 22 |
| hsa-miR-655-3p | ATAATACATGGTTAACCTCTTT | 22 |
| hsa-miR-130b-3p | CAGTGCAATGATGAAAGGGCAT | 22 |
| hsa-miR-382-3p | AATCATTCACGGACAACACTT | 21 |
| hsa-miR-424-5p | CAGCAGCAATTCATGTTTTGAA | 22 |
| hsa-miR-431-3p | CAGGTCGTCTTGCAGGGCTTCT | 22 |
| hsa-miR-2355-3p | ATTGTCCTTGCTGTTTGGAGAT | 22 |
| novel156_mature | CGCGGCGGCGGCGGCAGGCGC | 21 |
| hsa-miR-193b-5p | CGGGGTTTTGAGGGCGAGATGA | 22 |
| hsa-miR-138-5p | AGCTGGTGTTGTGAATCAGGCCG | 23 |
| hsa-miR-628-3p | TCTAGTAAGAGTGGCAGTCGA | 21 |
| hsa-miR-107 | AGCAGCATTGTACAGGGCTATCA | 23 |
| novel15_mature | GGGCGGAGGCGGGGGGAGA | 19 |
| hsa-miR-22-5p | AGTTCTTCAGTGGCAAGCTTTA | 22 |
| hsa-miR-660-5p | TACCCATTGCATATCGGAGTTG | 22 |
| hsa-miR-410-3p | AATATAACACAGATGGCCTGT | 21 |
| hsa-miR-148a-5p | AAAGTTCTGAGACACTCCGACT | 22 |
| hsa-miR-499a-5p | TTAAGACTTGCAGTGATGTTT | 21 |
| hsa-miR-15b-3p | CGAATCATTATTTGCTGCTCTA | 22 |
| hsa-miR-582-5p | TTACAGTTGTTCAACCAGTTACT | 23 |
| hsa-miR-382-5p | GAAGTTGTTCGTGGTGGATTCG | 22 |
| hsa-miR-485-3p | GTCATACACGGCTCTCCTCTCT | 22 |
| hsa-miR-29b-1-5p | GCTGGTTTCATATGGTGGTTTAGA | 24 |
| hsa-miR-532-5p | CATGCCTTGAGTGTAGGACCGT | 22 |
| hsa-miR-1185-1-3p | ATATACAGGGGGAGACTCTTAT | 22 |
| novel27_mature>novel75_mature | GCTAGGGATTGGCAAGGA | 18 |
| novel108_mature | CCCAGGGGAGCCCGGCGGGA | 20 |
| hsa-miR-450b-5p | TTTTGCAATATGTTCCTGAATA | 22 |
| hsa-miR-365b-3p>hsa-miR-365a | TAATGCCCCTAAAAATCCTTAT | 22 |
| hsa-miR-4485-3p | TAACGGCCGCGGTACCCTAA | 20 |

| Down-regulated miRNA_id | Sequence | Length |
| --- | --- | --- |
| hsa-miR-451a | AAACCGTTACCATTACTGAGTT | 22 |
| novel16_mature>novel18_mature | AGAGGGACGGCCGGGGGC | 18 |
| hsa-miR-629-5p | TGGGTTTACGTTGGGAGAACT | 21 |
| hsa-miR-615-3p | TCCGAGCCTGGGTCTCCCTCTT | 22 |
| hsa-miR-302b-3p | TAAGTGCTTCCATGTTTTAGTAG | 23 |
| novel64_mature | AGAGGCCGGCGGGAGCCG | 18 |

Table S1. After deep sequencing of Exos-IL6 and Exos-NC, 31 miRNAs were found to be upregulated and 6 miRNAs were downregulated in the Exos-IL6 group (fold change > 2, p < 0.05).
